# Effect of Tourist Activities on Fecal and Salivary Glucocorticoids and Immunoglobulin A in Female Captive Asian Elephants in Thailand

**DOI:** 10.3390/ani10101928

**Published:** 2020-10-21

**Authors:** Worapong Kosaruk, Janine L. Brown, Tithipong Plangsangmas, Patcharapa Towiboon, Veerasak Punyapornwithaya, Ayona Silva-Fletcher, Chatchote Thitaram, Jaruwan Khonmee, Katie L. Edwards, Chaleamchat Somgird

**Affiliations:** 1 Master’s Degree Program in Veterinary Science, Faculty of Veterinary Medicine, Chiang Mai University, Chiang Mai 50100, Thailand; woraph.kosa@gmail.com; 2 Center of Elephant and Wildlife Research, Faculty of Veterinary Medicine, Chiang Mai University, Chiang Mai 50100, Thailand; tithipong.pla@cra.ac.th (T.P.); towiboon@gmail.com (P.T.); chatchote.thitaram@cmu.ac.th (C.T.); jaruwan.khonmee@cmu.ac.th (J.K.); 3 Center for Species Survival, Smithsonian Conservation Biology Institute, Front Royal, VA 22630, USA; BrownJan@si.edu (J.L.B.); k.edwards@chesterzoo.org (K.L.E.); 4 Faculty of VeterinaryMedicine and Applied Zoology, HRH Princess Chulabhorn College of Medical Science, Chulabhorn Royal Academy, Lak Si, Bangkok 10210, Thailand; 5 Department of Food Animal Clinic, Faculty of Veterinary Medicine, Chiang Mai University, Chiang Mai 50100, Thailand; veerasak.p@cmu.ac.th; 6 Veterinary Public Health Centre and Food Safety for Asia Pacific (VPHCAP), Chiang Mai University, Chiang Mai 50100, Thailand; 7 Department of Clinical Sciences and Services, The Royal Veterinary College, Hawkshead Lane, Hertfordshire AL9 7TA, UK; ASilvaFletcher@rvc.ac.uk; 8 Department of Companion Animal and Wildlife Clinics, Faculty of Veterinary Medicine, Chiang Mai University, Chiang Mai 50100, Thailand; 9 Department of Veterinary Bioscience and Veterinary Public Health, Faculty of Veterinary Medicine, Chiang Mai University, Chiang Mai 50100, Thailand; 10 North of England Zoological Society, Chester Zoo, Upton-by-Chester, Chester CH2 1LH, UK

**Keywords:** Asian elephant, immunoglobulin A, glucocorticoids, saliva, feces, tourism, welfare

## Abstract

**Simple Summary:**

How tourist camp activities affect individual elephant welfare is an important and highly debated topic. Saliva and fecal samples were collected monthly for 1 year from 44 female Asian elephants that participated in three programs (saddle-, bareback-, or no-riding), and analyzed for glucocorticoids (GC) and immunoglobulin A (IgA). The hypothesis was that better welfare would be associated with low GC and high IgA concentrations. Both biomarkers showed significant variation with respect to camp size, riding activities, tourist-to-elephant ratios and seasonality, but not always consistently between feces and saliva, and not always in the predicted direction. However, there was no clear indication that riding per se negatively affected these two biomarkers. The lack of consistent responses highlights the difficulty in interpreting physiological data in relation to management factors, and suggests more work is needed to differentiate between potential chronic (feces) and acute (saliva) responses.

**Abstract:**

Asian elephants have been an important part of wildlife ecotourism in Thailand for over two decades. Elephants in tourist camps are exposed to a variety of management styles and daily activities that can potentially affect health and welfare. This study investigated relationships between a novel welfare biomarker, immunoglobulin A (IgA), and daily camp activities, and compared results to glucocorticoid (GC) measures. Often no-riding camps are portrayed as providing better welfare than camps that offer riding. Therefore, we predicted that elephants at no-riding camps would have lower GC and higher IgA concentrations, and a low GC/IgA ratio. Forty-four female elephants from six elephant camps were divided into three groups based on riding activities: saddle-riding, bareback-riding, and no-riding. Fecal and salivary samples were collected monthly for 1 year along with evaluations of body condition, foot health, and wounding. Camp environment and management varied among camps, although the major difference was in riding activities. Concentrations of GCs and IgA varied among the working groups, but not always consistently between sample matrices. Overall fecal glucocorticoid metabolite concentrations were lowest in the saddle-riding group. Only in one bareback-riding camp did the elephants exhibit a potentially positive welfare response with a low GC/IgA ratio over time. Other results varied between the two biomarkers, with considerable variability across camps, suggesting there is more to good welfare than whether elephants participate in riding or not. Several other human-induced stressors, like chaining, ankus use, and limited social opportunities are likely to be impacting well-being and should be considered to ensure management practices meet physical and psychological welfare needs.

## 1. Introduction

The Asian elephant (*Elephas maximus*) is an iconic species in Thailand, and one that has played an essential role in wildlife-based ecotourism for decades [1,2]. Today, there are approximately 3700 tamed Asian elephants working in tourist facilities (i.e., elephant camps) across Thailand [3,4]. The majority are located in the northern part of Thailand, in Chiang Mai province, which is considered the central hub of elephant ecotourism [1,5,6]. Elephants take part in a variety of tourist activities, like riding with or without a saddle, shows, feeding, and bathing [1,7]. While elephant trekking and riding are historically popular activities [1,8], recent criticisms have questioned whether they are harmful to elephant well-being [2]. Other concerns include elephants working long hours with inadequate rest periods, spending extended periods on chains, and being subjected to overuse of control equipment like the ankus (e.g., hook, bull hook, guide) [9]. As a result, a growing number of camps are now promoting more hands-off experiences, with tourists walking alongside elephants, feeding treats from behind a barrier, or just observing them from afar [6]. While limiting tourist interactions with elephants likely reduces the amount of control needed to keep animals and people safe, and thus may be better for welfare, new questions arise as to whether elephants are able to get adequate exercise and if diets are properly balanced [2,10].

Captive animal welfare is a broad concept that considers how management and husbandry practices meet physical and psychological needs [11], and relies on several tools to assess behavioral and physiological states [12]. Adrenal glucocorticoid (GC) activity is modulated by acute and prolonged stressors via the hypothalamic–pituitary–adrenal axis, and is the most common physiological indicator used to assess welfare in wildlife, including elephants [3,9,13,14]. In recent years, correlational studies have identified factors in the captive environment that affect welfare outcomes in elephants, including GCs [2,10]. In Thailand, elephants that participated in riding had better body condition and metabolic function (i.e., glucose, insulin, lipids) [7], and lower fecal GC metabolite (FGM) concentrations [3], perhaps related to the beneficial effects of exercise. However, saddle-riding also was associated with a higher prevalence of skin lesions [15], while higher wound scores due to misuse of the ankus were linked to increased GCs [3]. In India, FGM concentrations were lower in elephants used for tourist rides and patrolling compared to those that partook in an intensive and loud public festival [9]. Thus, the effects of tourist activities on elephant welfare are not entirely clear. It also is important to note that while commonly used as measures of stress, GCs also fluctuate under normal physiological conditions, and in elephants are related to sex [3,9], circadian rhythms [16], seasonal changes [17,18], the follicular phase in females [19] and musth in males [20]. For these reasons, difficulties in data interpretation can occur when GCs are used as the only welfare marker.

Recently, immunoglobulin A (IgA) has been proposed as a novel biomarker for welfare assessments of wildlife species [21,22,23], including Asian elephants [16,24]. IgA is the primary immune protein in humoral mucosal immunity as first-line mucosal protection against pathogens [25] and is an indicator of health and immune function [25,26]. In one Asian elephant, IgA was significantly elevated in association with a systemic illness [24]. However, in other species, increased IgA was also observed in response to positive stimuli, like environmental enrichment [25], and relaxing activities like watching movies, lying down, and massage [27]. Because chronic stress can suppress immune function, a decrease in IgA may indicate poor welfare conditions, whereas high concentrations could indicate a positive welfare state [25,28]. Thus, measures of IgA may be useful in determining how animals respond to different types (positive or negative) and durations (acute or chronic) of stressful stimuli, both intrinsic and extrinsic [16,25]. Furthermore, combining the two indices could provide more robust interpretations of findings from welfare studies [24]. For example, inverse relationships between GC and IgA have been found in several species [21,29,30,31], so examining GC to IgA ratios might be even more informative. Both biomarkers can be measured non-invasively in feces and saliva [16,24,25], which adds to their utility by avoiding unintentional stress due to blood draws [9,25,32], but more work is needed to determine how they function in the stress response of elephants.

This study is a first step in trying to understand how captive elephants respond to different human-imposed stressors in the form of feeding, bathing, and riding. Because no-riding camps are often portrayed as providing better welfare than camps that do offer riding, the goal was to examine how GC and IgA varied among elephants at six tourist camps in Thailand, particularly in relation to riding activities. If riding is a significant stressor, we would expect to observe lower GC and higher IgA concentrations in elephants at no-riding camps. We also examined how these biomarkers related to other camp management factors, and to scores of body condition, foot health and wounding. Results could provide useful information and a new viewpoint to the use of IgA as a welfare measure, and identify how management practices affect the overall well-being of working elephants in Thailand.

## 2. Materials and Methods

### 2.1. Animal Ethical Consent

This study was approved by the Faculty of Veterinary Medicine, Chiang Mai University (CMU), Animal Care and Use Committee (Number S38/2561).

### 2.2. Study Animals

Information on each of the six camps is summarized in Table 1. All were working elephant tourist camps in Mae Rim and Mae Taeng districts, Chiang Mai province, Thailand (18.7883° N, 98.9853° E). Subsets of elephants were selected by each camp owner based on them having a cooperative mahout for sample collection and a subjective tame disposition. A total of 44 female Asian elephants, 7–60 years of age (mean, 31.6 ± 2.1 years) were evaluated. The age data of participated elephants (Table S2) were excluded from the published information. All elephants were provided fresh roughages (e.g., grass, corn stalks) as the main food source 4–6 times/day, with tourists allowed to feed bananas and sugar cane (~20–30% of the total diet; camp veterinarian estimation) throughout the workday at four of the six camps. Veterinary care was provided by camp veterinarians or veterinary assistants, and all participating elephants were deemed healthy.

Elephants were divided into three groups based on work activities: riding with and without a saddle, and no-riding. For saddle-riding (14 elephants), elephants carried one or two tourists in a wooden saddle (saddle weight ~30 kg) with a mahout on the neck. Elephants walked 20–45 min along a fixed trail in the jungle, with water (i.e., water tanks, river) provided along the way. Both saddle camps organized elephant rides as rounds (~30–90 min/round; 1–2 rides per round; 4 rounds/day; ~4–8 rides/day). Once each round was completed, elephants were given a 1 h break before beginning the next round. During the rest period, elephants were chained and offered browse. After the final round (~15:00 h), mahouts took elephants to bathe in the river before chaining them overnight with food. Supplemental feeding was not scheduled for riding elephants at the two saddle-riding camps.

For bareback-riding (11 elephants), both camps allowed one or two tourists to sit on the neck with a mahout following on foot. Both camps had half- and full-day programs. Half-day programs provided ~3 h of activities (riding, feeding, bathing) twice a day (once in the morning, once in the afternoon). The full-day program lasted ~4 h, with additional activities like making sticky rice balls to feed the elephants, and spending more time on the ground with them (1 h or more compared to the half-day program). In both programs, riding took place along a jungle trail for around 25–35 min. Tourists participated in activities such as feeding bananas and sugar cane (both camps), bathing in a river (Camp C, splashed water; Camp D, active scrubbing with commercial shower cream), and applying mud to the elephant’s skin (head and body) in a mud wallow (Camp C). Free foraging was allowed throughout the day along the jungle trail. In the half-day program, elephants were chained during the break and offered food (Camp C, 11:00–14:30 h; Camp D, 11:00–15:00 h), whereas elephants in the full-day programs were unchained and allowed to socialize and forage in the forest for 1 h (12:00–13:00 h). Elephants returned from the jungle after work (half-day, ~17:30 h; full-day, ~16:00 h) and were chained overnight with food provided until 7:00 h.

In the non-riding group (19 elephants), both camps provided half-day and full-day programs with 3 h (twice a day) and 4 h (once a day) of activities, respectively. Elephants were free to roam unchained with conspecifics in a grassy field area enclosed with (Camp F) or without (Camp E) a steel fence. For the first 30 min of each program, staff provided general information about elephants while tourists observed from afar. After that, tourists began their activities, which included feeding and touching with (Camp F) or without (Camp E) a protective barrier between elephants and tourists, walking, applying mud to the elephant’s body (Camp E), and bathing (both) in a river by actively scrubbed with herbal (*Entada rheedii*) vines (Camp F) or commercial shower cream (Camp E, F). The additional activities of the full-day program included sticky rice ball making and feeding, paper-making from elephant dung, and extended time to spend with elephants. Elephants at these camps spent most of the time free-roaming with mahouts nearby in open dirt areas with access to a water source (pond or river). At Camp F, elephants were free-roaming during breaks away from tourist activities (half-day, 11:00–14:30 h; full-day, 12:00–13:00 h), where at Camp E, elephants were chained during break time (half-day, 11:00–1500 h; full-day, 12:00–13:00 h); both offered food during the breaks. Elephants were chained overnight after tourist activities (half-day, ~17:30 h; full-day, ~16:00 h) until 7:00 h.

### 2.3. Sample Collection

Fecal (*n* = 525) and salivary (*n* = 521) samples were collected monthly from January to December 2019 between 9:00 and 12:00 h, although some saliva data are missing due to sampling constraints. Fresh fecal samples were collected on the same day as saliva. The fecal ball was mixed by hand and two ~20 g subsamples were placed into two zip-lock plastic bags and frozen at −20 °C for separate GC and IgA extraction processes. Saliva was collected using a Salivette^®^ kit (Sarstedt Inc., Newton, NC, USA) [16,24] by wiping a synthetic swab inside the buccal area for 30–60 s; collection was complete within 5 min or less. The mahout used food to get the elephant to open its mouth so the sample collector could swipe the mouth with a gloved hand, and rewarded it after collection was complete. Samples were kept in a 4 °C cool box for <8 h until centrifugation at 1500× *g* for 5 min at 15 °C. Two swabs were collected, and the saliva was pooled for an average volume of 500 µL (100–1500 µL) per sample. Saliva was stored frozen at −20 °C until analysis.

### 2.4. Physical Scoring Factors

Body condition (BCS), foot health (FS), and skin wound (WS) scores were assigned to each elephant at the time of sample collection. The 5-point BCS scale was based on Morfeld et al. [33] and adapted for use in Asian elephants in Thailand by Norkeaw et al. [3]; 1 indicates the thinnest and 5 indicates the fattest score. The FS was based on Bansiddhi et al. [3], with 0 = no foot or nail problems to 3 = severe foot problems. The WS was developed by Schein et al. [34] and adapted in Thailand by Bansiddhi et al. [3], where 0 represents no wounds and 2 indicates major wounds.

### 2.5. Fecal Extraction

#### 2.5.1. Fecal Extraction for GC Analysis

Fecal samples were extracted following the procedure of Bansiddhi et al. [3]. In brief, frozen fecal samples were thawed at room temperature (RT) and placed in a conventional oven (60 °C) for 24–48 h or until the samples were dry. Powdered feces were mixed and ~0.1 g (±0.001 g) placed into glass tubes and then 4.5 mL of EtOH and 0.5 mL of distilled water were added and vortexed briefly. Samples were extracted twice, first by boiling in a water bath (90 °C) for 20 min, with 95% EtOH added to keep the volume at 5 mL. Samples were centrifuged at 960× *g* for 20 min and the supernatant was decanted into labeled tubes. Another 5 mL of 90% ethanol was added to the pellet, which was then vortexed briefly and centrifuged at 960× *g* for 20 min. The fecal extracts were combined and dried in a 90 °C water bath. Fecal extracts were re-suspended in 3 mL of 95% EtOH, dried down again, and finally re-suspended in 1 mL of 50% methanol. Samples were stored at −20 °C until analysis.

#### 2.5.2. Fecal Extraction for IgA Analysis

The fecal extraction protocol for IgA analysis was adapted from Edwards et al. [24]. Frozen fecal samples were dried in a lyophilizer at −40 °C for 24–48 h, and then 0.1 g (±0.001 g) of crushed, mixed sample was extracted with 3 mL of phosphate-buffered saline (PBS) with Tween (PBS-T; 0.01 M phosphate buffer, 0.50 M NaCl, 0.1% Tween 20^®^, pH 7.2) by vortexing overnight on a multi-tube pulse vortexer (Glas-Col, Terre Haute, IN, USA), with a motor speed of 50 and pulse ON. Samples were then vortexed briefly and centrifuged at 1800× *g* for 20 min at 4 °C. The supernatant was decanted into a clean tube and centrifuged again at 3500× *g* for 10 min at 4 °C to remove any remaining particulates. It was then decanted into a clean tube, dried in a lyophilizer at −40 °C overnight, and re-suspended with 0.5 mL ultra-purified water. Samples were stored at −20 °C until analysis.

### 2.6. Enzymeimmunoassays

FGM concentrations were measured in fecal extracts diluted 1:3 in assay buffer using s double-antibody EIA with a polyclonal rabbit anti-corticosterone antibody (CJM006, Coralie Munro) validated for Asian elephants in Thailand [7]. Samples and corticosterone standards (50 µL) were added to wells in duplicate followed by corticosterone-HRP (25 µL; 1:30,000) and then the anti-corticosterone antibody (25 µL; 1:100,000). Plates were incubated at RT for 2 h before adding 100 µL of TMB solution, followed by incubation for 20–35 min, and addition of the stop solution (50 µL). Incubations were conducted in the dark. The absorbance was measured at 450 nm by a microplate reader (TECAN, Männedorf, Switzerland). Assay sensitivity was 0.099 ng/mL. Intra- and inter-assay coefficients of variation (CV) were <10 and 11.7%, respectively.

Salivary cortisol was measured by a double-antibody enzyme immunoassay (EIA) validated for elephants [16] that uses a polyclonal rabbit anti-cortisol antibody (R4866, Coralie Munro, University of California Davis, Davis, CA, USA). Microtiter plates (Nunc^TM^, F96 Maxisorp immune plate, Roskilde, Denmark) were pre-coated with anti-rabbit IgG (150 µL; 10 µg/mL) per well as described by Plangsangmas et al. [16]. Cortisol standards (50 µL; 0.078–20 ng/mL) and salivary samples (50 µL; neat) were added in duplicate followed by cortisol-horseradish peroxidase (HRP) (25 µL; 1:16,000) immediately added to each well, except for non-specific binding wells. The primary anti-cortisol antibody (25 µL; 1:75,000) was added to all wells and incubated for 1 h at RT. Plates were washed four times with wash buffer (1:20 dilution, 20× wash buffer; Cat. No. X007, Arbor Assays, MI, USA) to remove unbound components. TMB substrate solution (KPL TMB Microwell Peroxidase Substrate System 2-contents) (100 µL) was added and the plates were incubated for 5 min at RT, followed by the addition of stop solution (50 µL). Absorbance measured at 450 nm. Assay sensitivity (based on 90% binding) was 0.11 ng/mL. Intra- and inter-assay coefficients of variation (CV) were <10 and 10.7%, respectively

IgA in fecal and salivary samples was measured using commercially available components as described by Edwards et al. [24] with some modifications as described by Plangsangmas et al. [16]. In brief, a polyclonal rabbit anti-human IgA antibody (A0262, Dako, Glostrup, Denmark) was diluted in PBS (0.01 M phosphate buffer, 0.15 M NaCl, pH 7.2) to a 1 µg/mL working solution and then added to 96-well microtiter plates (100 µL/well). After incubation at 4 °C overnight, PBS-T was used to aspirate and wash each plate three times. Human colostrum IgA was used as the standard (0.039–100 ng/mL) and high- and low-quality control samples. Fecal and salivary extract samples were diluted with PBS-T as needed (saliva 1:50–600 and fecal extract 1:4–30). Plates were incubated at RT for 2 h on a plate shaker set to 150 rpm before washing with PBS-T again. Polyclonal rabbit anti-human IgA antibody conjugated with HRP was diluted in PBS-T 1:2500 and added at 100 µL/well. Plates were incubated on a plate shaker (150 rpm) before a final wash. TMB (100 µL) was added to each well and incubated in the dark for 20 min at RT. After this, 50 µL of 2M H_2_SO_4_ was added to stop the reaction. Optical densities were determined at 450 nm by a microplate reader (TECAN). The EIA was validated for fecal IgA in this project by showing parallelism between serial dilutions of fecal extracts and the standards (y = 2.16x + 1.45, R^2^ = 0.97), and significant recovery of fecal IgA added to a low concentration sample before analysis (y = 1.11x − 0.69, R^2^ = 0.99). Assay sensitivity was 0.46 ng/mL. The intra- and inter-assay CVs were <10 and 12%, respectively.

### 2.7. Statistical Analysis

R statistical software version 3.5.1 was used to conduct all statistical analyses in this study without transforming the data. Normality and variance of the data were examined by QQ plot (R package: Quantile-Quantile plot 0.0.4; qqplotr [35]). Mean data for IgA and GC concentrations, and BCS, FS, and WS are shown as a mean ± standard error of the mean (SEM). Repeated measures data were analyzed using the Generalized Least Squares method (GLS, R package: non-linear mixed effect model 3.1–148; nlme [36]) to determine the effects of each variables (i.e., elephant activities, time, and camp management) independently on GC and IgA concentrations. Both GC and IgA were analyzed with regards to the three major seasons in Thailand: summer (16 February–15 May), rainy (16 May–15 October) and winter (16 October–15 February) (Northern Meteorological Center, Meteorological Department, Ministry of Information and Communication Technology, Chiang Mai, Thailand), and also the high (November–February) and low (March–October) tourist seasons (Tourism Authority of Thailand) as described by Norkeaw et al. [4]. Differences in means between environmental and tourist seasons were analyzed using a univariable GLS model. Mean GC to IgA ratios were calculated within the same biological sample and differences analyzed by GLS. Generalized Estimating Equations (GEE, R package: Generalized Estimating Equation Package 1.3-1; ggpack [37]) were conducted to analyze repeated measures data for BCS, FS, and WS among camp. Tukey post-hoc tests (R package: least-squares means 2.30-0; lsmeans [38]) were further used to analyze differences in mean GC and IgA concentrations between activity groups and months. Repeated measures correlations (R package: repeated measure correlation 0.3.1; rmcorr [39]) were used to determine relationships between individual GC and IgA concentrations in feces and saliva.

## 3. Results

### 3.1. Tourist Camp Activities

Tourist camp activities are summarized in Table 1. Facilities were categorized based on elephant numbers as described by Bansiddhi et al. [6]: three camps (Camp A, B, F) were large with >30 elephants, two camps were medium size (10–30 elephants), and one was considered small (<10 elephants, Camp D). The two saddle-riding camps had a higher male to female ratio (Camp A, 0.75; Camp B, 0.44) followed by no-riding (Camp F, 0.38; Camp E, 0.31) and bareback-riding (Camp C, 0.05; Camp D, 0.25) camps. The median tourist-to-elephant ratio was categorized as high (>3, Camp A), moderate (2–3, Camp B, D, E), or low (<2, Camp C, F). All camps chained elephants for the vast majority of the time, 3–4 h during the day and 16–18 h overnight; Camp F, a large camp, chained elephants less during the day at 0–2 h.

### 3.2. FGM and Salivary Cortisol Measures

Summary statistics for fecal and salivary GC data are shown in Table 1. The monthly mean of GC concentrations in each elephant activity is provided in Supplementary Materials Table S1, monthly individual GC plots (Figure S1) were not in published resources

For FGM, elephants at Camp F had the highest, while those at Camp A had the lowest concentrations. Overall, those used for saddle-riding (28.4 ± 1.07 ng/g) had lower FGM concentrations than for bareback (36.2 ± 1.78 ng/g) or no-riding (36.7 ± 1.32 ng/g) (*p* < 0.01), with no difference in FGM between bareback and no-riding elephants (*p* = 0.97). FGM concentrations differed (*p* < 0.01) in relation to high (25.8 ± 1.27 ng/g), moderate (32.5 ± 1.13 ng/g), and low (38.0 ± 1.33 ng/g) tourist-to-elephant ratios. There also was a difference in FGM concentrations for total chain hours, being lower for less (<17 h, 39.2 ± 1.60 ng/g) compared to more (>17 h, 31.7 ± 1.04 ng/g) chaining time (*p* < 0.01). More social time was associated with higher FGM concentrations (*p* < 0.01) across 0-h (28.4 ± 1.07 ng/g), 1-h (34.4 ± 1.31 ng/g), and 2-h (39.2 ± 1.72 ng/g) groups, as was tourist feeding (36.5 ± 1.02 ng/g) compared to no feeding (28.4 ± 1.50 ng/g) (*p* < 0.01).

**Figure 1:**
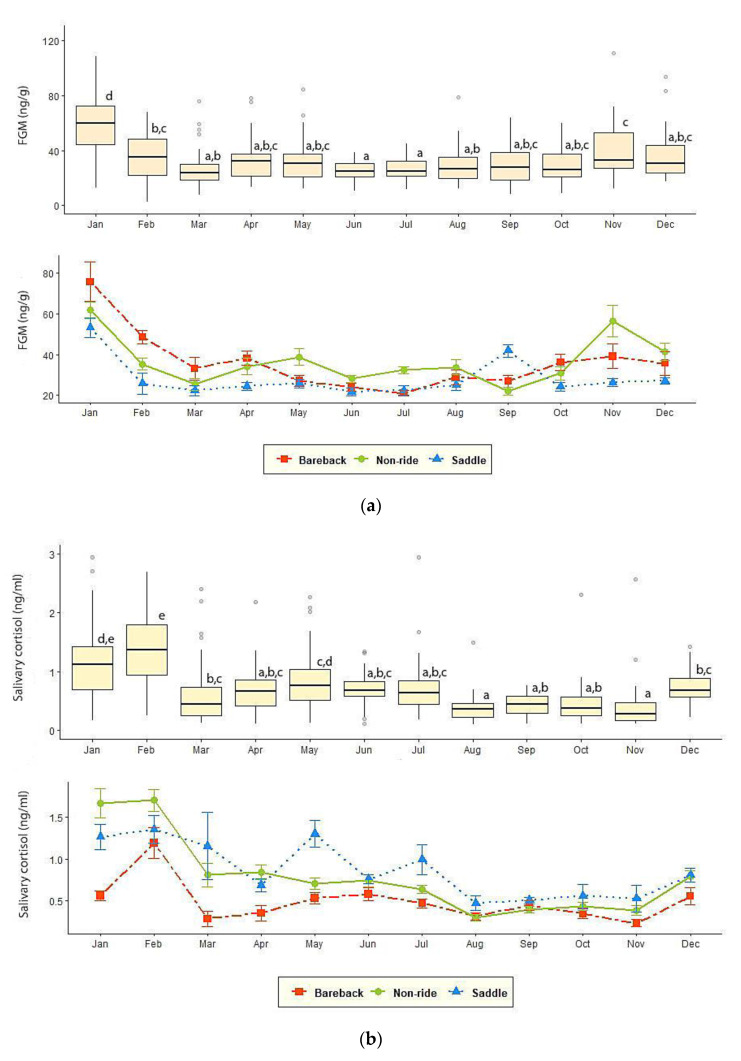
Mean (± SEM) monthly (**a**) fecal glucocorticoid metabolite (FGM) and (**b**) salivary cortisol concentrations in elephants involved in saddle-riding, bareback-riding, or no-riding. Box plots illustrate salivary cortisol and FGM measures for all elephants combined (N = 44). Whiskers represent median, quartiles, and the 25th/75th percentiles, error bars represent the 10th/90th percentiles, and open circles indicate outliers. Different superscripts indicate a significant difference at *p* < 0.05.

Across environmental factors, the highest concentration was observed in January (62.40 ± 3.51 ng/g), the lowest in June (25.10 ± 1.11 ng/g), and between March and July concentrations were generally low (Figure 1). Overall FGM concentrations were highest in the winter (43.9 ± 1.82 ng/g) compared to the rainy (29 ± 0.92 ng/g) and summer (28.8 ± 0.99 ng/g) seasons (*p* < 0.01), the latter two of which were not different (*p* > 0.05). FGM concentrations also differed between high (43.9 ± 1.82 ng/g) and low (28.9 ± 0.67 ng/g) tourist seasons (*p* < 0.01).

For salivary cortisol, results often contrasted with FGM. For example, Camps A and F had the highest, while Camp D had the lowest measures. Elephants that participated in a bareback-riding program (0.49 ± 0.05 ng/mL) exhibited lower salivary cortisol concentrations than those in saddle- (0.86 ± 0.04 ng/mL) and no-riding (0.78 ± 0.03 ng/mL) groups, with the latter two being the same (*p* = 0.31). Additionally, in contrast to feces, where there were no differences, salivary cortisol varied based on camp size, with large camps having the highest salivary measures (0.85 ± 0.04 ng/mL) compared to medium (0.59 ± 0.03 ng/mL) and small (0.44 ± 0.07 ng/mL) camps. In those with a high tourist-to-elephant ratio (0.89 ± 0.07 ng/mL), salivary cortisol was higher compared to low ratio camps (0.69 ± 0.03 ng/mL, *p* < 0.05). Salivary measures also differed between camps that provided 1-h of free time to socialize (0.57 ± 0.04 ng/mL) compared to 0-h (0.86 ± 0.04 ng/mL) and 2-h (0.83 ± 0.05 ng/mL), the latter two of which were not different. Camps with scheduled tourist feeding had elephants with lower salivary cortisol concentrations (0.68 ± 0.03 ng/mL) compared to camps that did not (0.86 ± 0.05 ng/mL, *p* < 0.01).

Salivary cortisol followed a seasonal pattern similar to feces, with the highest concentrations in January and February (1.26 ± 0.11, 1.46 ± 0.09 ng/mL) and the lowest between August (0.36 ± 0.04 ng/mL) and November (0.39 ± 0.06 ng/mL), with more variable concentrations between March and July (Figure 1). Concentrations also varied across environmental seasons, being highest in the winter (0.96 ± 0.05 ng/mL), intermediate in the summer (0.75 ± 0.04 ng/mL) and lowest in the rainy (0.49 ± 0.03 ng/mL) season. There also was a difference between high (0.96 ± 0.05 ng/mL) and low (0.63 ± 0.03 ng/mL) tourist seasons (*p* < 0.01).

Based on the univariable GLS model, significant factors that affected FGM included work activities, tourist-to-elephant ratio, work hours, chain hours, socialization time, tourist feeding and tourist and environmental seasons, while for salivary cortisol, type of work, camp size, tourist-to-elephant ratio, socialization time, tourist feeding, and seasons were significant (Table 2). There was no age effect on camp activities and GC measures.

### 3.3. Fecal and Salivary IgA

Descriptive statistics for fecal and salivary IgA data are displayed in Table 1. The monthly mean of IgA concentrations in three elephant activity groups is given as Supplementary Materials in Table S1. Monthly IgA measures in each elephant (Figure S2) were not provided in published resources.

Camp C had the highest fecal IgA concentrations among camps. Overall, elephants in a bareback-riding program had higher fecal IgA concentrations (2.02 ± 0.18 µg/g) compared to saddle (0.88 ± 0.16 µg/g) and no-riding (0.77 ± 0.14 µg/g) programs (*p* < 0.01), with no difference between the latter two (*p* = 0.86). Medium (1.65 ± 0.10 µg/g) camps had the highest fecal IgA concentrations, with large (0.85 ± 0.06 µg/g) (*p* < 0.01) and small (0.69 ± 14 µg/g) camps having similar values. Lower concentrations of fecal IgA were found in elephants at camps with a high (0.68 ± 0.09 µg/g) versus low (1.42 ± 0.08 µg/g) tourist-to-elephant ratio (*p* < 0.05). Fecal IgA concentrations also differed between camps that provided 1 h of socialization time (1.54 ± 0.09 µg/g) compared to 0 h (0.88 ± 0.08 µg/g) and 2-h (0.82 ± 0.08 µg/g), the latter two of which were similar.

**Figure 2:**
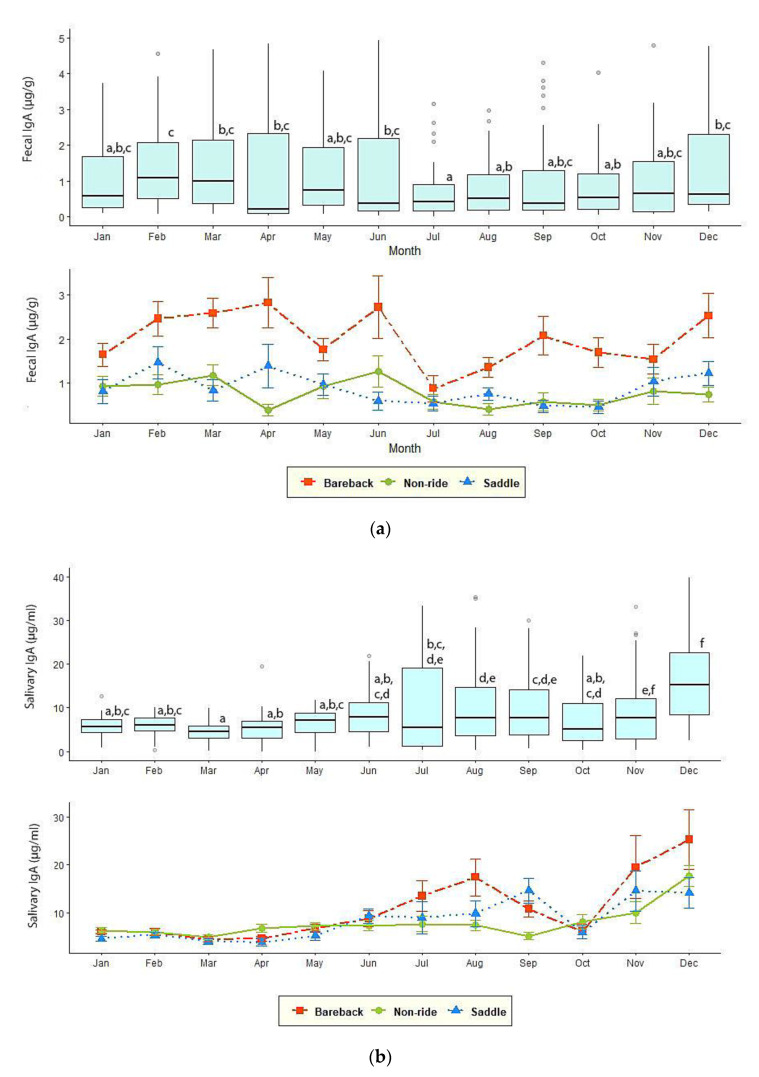
Mean (±SEM) of (**a**) fecal (µg/g) and (**b**) salivary (µg/mL) IgA concentrations in each elephant activity group. Box plot showed overall fecal and salivary IgA measures in combination with three elephant groups (N = 44). Whiskers represent median, quartiles, and the 25th/75th percentiles, error bars represent the 10th/90th percentiles, and open circles indicate outliers. Different superscripts indicate a significant difference at *p* < 0.05.

IgA varied throughout the year (*p* < 0.01), but the patterns differed from those of GC (Figure 2). Monthly fecal IgA concentrations were variable, with the highest in February (1.50 ± 0.19 µg/g) and lowest in July (0.64 ± 0.11 µg/g, *p* < 0.01), and no clear seasonal pattern. Fecal IgA concentrations also differed between high and low tourist seasons (1.23 ± 0.09, 1.06 ± 0.06 µg/g, *p* < 0.05), while the rainy season showed the lowest concentrations (0.79 ± 0.07 µg/g) compared to the summer (1.33 ± 0.11 µg/g) and winter (1.23 ± 0.09 µg/g) (*p* < 0.01).

Similar to feces, camp C had high salivary IgA measures together with Camp B. In contrast to feces; however, salivary IgA was not affected by work type (saddle, 8.34 ± 1.11 µg/mL; bareback, 10.74 ± 1.25 µg/mL; no ride, 7.88 ± 0.95 µg/mL) or camp size (small, 3.36 ± 0.41 µg/mL; medium, 10.4 ± 0.79 µg/mL; large, 8.21 ± 0.43 µg/mL). Lower concentrations of salivary IgA were found in elephants at camps with a high (5.65 ± 0.52 µg/mL) versus low (9.73 ± 0.58 µg/mL) tourist-to-elephant ratio (*p* < 0.05).

The annual pattern of salivary IgA differed somewhat from feces, with low concentrations for the first 5 months of the year before increasing to a peak in December (18.3 ± 2.11 µg/mL). Salivary IgA concentrations differed between high (10.8 ± 0.88 µg/mL) and low (7.69 ± 0.36 µg/mL) tourist seasons (*p* < 0.01), similar to fecal measures. However, unlike feces, elephants exhibited the lowest concentrations in summer (6.14 ± 0.29 µg/mL) compared to the rainy (9.28 ± 0.64 µg/mL) and winter (10.8 ± 0.88 µg/mL) seasons (*p* < 0.01).

Based on the univariable GLS model, significant factors related to fecal IgA were work activities, camp size, tourist-to-elephant ratio, socialization time, and tourist and environmental seasons, while those for salivary IgA concentrations included tourist-to-elephant ratio, and tourist and environmental seasons (Table 2). There was no age effect on camp activities and IgA measures.

### 3.4. Variable Relationships

Descriptive statistics for BCS, FS, and WS are shown in Table 1. The BCS mode was 4, with no elephants scoring a 1 or 2. From the GEE, BCS was higher in both no-riding camps (Camp E, F) and one bareback-riding camp (Camp C) compared to those where elephants participated in other camps (*p* < 0.05). The majority of elephants (70.96%) had a FS of 1, mostly related to nail cracks. No elephants had a FS = 3. Both bareback-riding camps had better (i.e., lower) FSs among the six camps (*p* < 0.05). No elephants had a WS = 2; the majority (83.53%) scored a 0. Overall, saddle-riding elephants had a higher average WS than those in the bareback-riding (*p* < 0.01) and no-riding (*p* < 0.01) programs. The majority with a WS = 1 were in a saddle program, and wounds were generally related to ankus use, with lesions apparent on the forehead; only one elephant had lesions related to saddle equipment (Camp A). However, one bareback-riding camp (Camp D) had a WS that was comparable to the saddle-riding elephants, which again was related to ankus use that caused cuts and abrasions. The other bareback-riding camp (Camp C) and both no-riding camps had the fewest skin wounds, with >90% scoring 0.

There were no differences in FGM, fecal or salivary IgA concentrations between the BCS categories (*p* > 0.05), except for salivary cortisol concentrations that were higher in elephants with a BCS = 3 (0.85 ± 0.13 ng/mL) compared to BCS = 5 (0.33 ± 0.14 ng/mL, *p* < 0.05), but not with a BCS = 4 (0.76 ± 0.05 ng/mL, *p* > 0.05). For FS, only salivary IgA differed between FS = 0 (12.49 ± 1.24 µg/mL) and FS = 1 (7.88 ± 0.87 µg/mL) or FS = 2 (5.76 ± 2.59 µg/mL, *p* < 0.01), with no differences between the latter two (*p* > 0.05). GC and IgA concentrations were not affected by WSs (*p* > 0.05).

Ratios between GC and IgA within the same biological samples were calculated and averaged by elephant (Table 1 and Table 3). Camp D had the highest fecal GC/IgA ratio among the six facilities, while Camp B and C had the lowest. However, for saliva, Camp A had the highest while Camp C had the lowest ratio. Overall, bareback-riding elephants presented a lower GC/IgA in feces compared those participating in the other activities (*p* < 0.01), but not with saliva. Camp size also affected only the fecal ratio, with elephants in small camps (but only camp D) having a higher ratio compared to medium and large camps. By contrast, the tourist-to-elephant ratio affected only the salivary GC/IgA ratio, with a high tourist-to-elephant ratio having higher GC/IgA compared to moderate and low ratios. The GC/IgA ratio did not differ across environmental and tourist seasons or the WS, but was affected by BCS = 3 having the highest ratio in both matrices, and FS = 2 having a higher ratio in feces.

Repeated measures correlations between the two biomarkers in feces and saliva, and relationships between physical exam parameters are displayed in Table 4. A weak positive correlation was found between FGM and salivary cortisol concentrations (*p* < 0.01); however, no other significant correlations between GCs and IgA were found.

## 4. Discussion

This is the first study to examine GCs and IgA in combination throughout the year in Thailand tourist camp elephants. While considerable work has been done using GCs as a welfare marker, including in Asian elephants [3,7,14], few have examined IgA in relation to environmental, management, and welfare factors in this species. We found several management and work activities, particularly with respect to riding, affected GC and IgA concentrations, in addition to several physical welfare measures. Our hypothesis was that positive welfare states would be associated with lower GCs and higher IgA, and interestingly the lowest GC/IgA ratios were found in elephants at one of the bareback-riding camps. However, clear or consistent relationships among the other camp variables were lacking, which suggests there is more to welfare than whether elephants participate in riding or not. Finally, for some variables there was agreement between sample matrices, but not all, creating further difficulty in interpreting biological responses to some of the management practices.

FGM results were comparable to our previous studies [3,7], where elephants that participated in saddle-riding had overall lower FGMs than those involved in bareback or no-riding activities. It is important to note; however, that four of the six camps were involved in those studies, although not all elephants were the same. Three of nine elephants in Camp A, two of five in Camp B, one in Camp C, and three in Camp D had been evaluated before. Nevertheless, all sample collection was contemporary and showed remarkable consistency with respect to FGM and riding activities. This finding emphasizes again that exercise likely is beneficial to the physiological health of captive elephants [3,4,40,41], and perhaps can counteract the negative effects of tourist feeding. Elephants at camps that limit exercise and where tourists are allowed to feed supplements, such as bananas and sugar cane, are more likely to be obese and exhibit metabolic derangements [7,10], and in this study, the two no-riding camps had the highest average BCSs. High energy food consumption can cause adrenal activation given a primary function of GCs is energy mobilization [14]. Romain et al. [42] also noted digestible energy intake was higher than recommended in their study of one Thai tourist camp. Finally, in zoo primates, there was a positive relationship between dietary carbohydrates, sugars, glucose, and fruits and cortisol [43]. Thus, as more camps switch to less intensive tourist activities and eliminate or limit riding, a trend we have observed in northern Thailand [6], it will be even more important to provide opportunities for elephants to be physically active and to limit the amount of high calorie supplements. More time off chains would allow elephants to be more active on their own, and increase their ability to socialize and free-forage.

While saddle-riding elephants had the lowest FGM, the lowest salivary cortisol was observed in the bareback-riding elephants. One possible explanation for the difference may be the more invasive nature of saliva collection compared to fecal sampling [44]. Even though saliva collection took less than 5 min, the elephant could see the researcher approaching from a distance, and then restraint (mahout commands or ankus use) was generally used during collection. All of these could have caused an acute stress response within the ~10 min estimated lag period from circulation to secretion [45]. By contrast, FGM represents an accumulation of GCs over a period of ~36 h [46] and so could provide a more steady-state assessment of adrenal activity. Although empirical data are lacking, one possible explanation for lower salivary cortisol in the bareback-riding group may be that for tourist safety, the elephants are more docile and accepting of human interactions, and thus less responsive to saliva collection. By comparison, the restricted use of basic restraint equipment (i.e., ankus) at no-riding camps, or conversely the heavy use at saddle-riding camps might have led to higher concentrations because elephants were less comfortable with restraint and sampling. Not all elephants are well-trained to open their mouths on command, which also could have been a factor in the different GC responses. For these reasons, assessment of adrenal activity via salivary cortisol may be useful only if elephants are well conditioned to the collection procedure. In cattle, animals that are regularly handled become progressively more docile in the face of potential stressful procedures [47]. Several studies further highlight the importance of temperament when assessing GC activity, with calmer, less reactive traits being associated with lower basal or post-stimulation cortisol concentrations [48,49,50]. In cattle, Grandin [51] proposed that rough handling may be more stressful to temperamental animals than those that are calmer. Furthermore, more reactive breeds of cattle and sheep may show more agitated behavior after handling, transport, or restraint along with higher cortisol [52,53]. Thus, additional studies are clearly warranted to assess how elephant temperament and past experience may be affecting adrenal responsiveness to human activities and sample collection procedures, and how those are reflected in fecal versus salivary measures.

In examining how the tourist-to-elephant ratio affected GCs, we found an inverse relationship for feces and saliva, such that more tourists per elephant were associated with lower FGM. This was unexpected, given studies in other species that show higher visitor numbers are associated with increased GC activity in many zoo animals [54,55,56], although not always [57,58]. Camp size also did not affect FGM, although for saliva, elephants in larger camps did exhibit higher concentrations. Such discrepancies between fecal and saliva measures make it difficult to understand the exact nature of some of these relationships [59]. Considering circulation to excretion lag times, differences may be related to assessment of short-term (saliva, ~10 min) versus long-term (feces, ~36 h) GC activity, which is why analyzing both may be more informative depending on the question [60]. Finally, the limits of using GCs as a sole measure of stress and welfare are well known, not the least of which is that they are released in response to both positive and negative stimuli [44], which is why IgA also was measured in this study as a possible welfare marker.

For IgA, there was considerable variability across individuals, comparable to earlier reports in elephants [16,24] and other species [61,62,63]. While most studies have used saliva [54,55,56,57], IgA can be measured in feces, including in elephants [16,24], which provides a noninvasive way to use this immune biomarker. Bareback-riding elephants exhibited higher fecal IgA concentrations overall, although that was primarily due to elephants at Camp C. Camp B in the saddle-riding group also had comparatively higher fecal IgA compared to the other camps. By contrast, there were no differences in salivary IgA concentrations across the work activity types. As both fecal and salivary IgAs are secretory products of mucosa, we expected patterns to be similar between the two matrices. However, again, differences in lag times could be reflecting acute versus chronic measures of immune function and therefore welfare. In humans, Kawano et al. [64] did not find differences in salivary IgA between workers who participated in highly stressful compared to less stressful jobs, perhaps because the former had learned to adjust and cope. Birkett et al. [65] showed that concentrations of salivary IgA in humans returned to baseline within 20 min of stress exposure. Our collection protocol was only once a month, so our IgA results may not reflect immune changes in response to more acute activities. A more frequent sampling protocol (i.e., before and after a stimulus) in association with challenge tests (e.g., acute maximal exercise [66], Trier Social Stress Test [65]) may be required to assess how specific factors affect IgA production, as has been done in humans [62,65,66,67,68]. Both bareback-riding camps in our study provided foraging opportunities during tourist activities, which might mitigate stress [69,70], but higher IgA potentially related to better welfare was only observed in Camp C. As with FGM, management factors other than or perhaps in addition to riding likely play a role in IgA production, so it is unclear if higher IgA would be a sign of positive welfare and healthy immune function in this context. Ultimately using IgA as a welfare marker is complicated by the fact that chronic stressors also can suppress immune function and reduce IgA production [25,28], while acute illnesses that elicit immune responses to cope with underlying pathology can increase it [24,71,72]. Thus, interpretation of IgA measures, just like GCs, is not always straightforward, as both can increase in response to acute stressors of a non-immune nature [27,73]. In addition, as with other potential indicators of well-being, it is important to understand normal physiological levels both within and between individuals, as well as in response to specific events. More work is also needed to understand how GCs and IgA interact and respond to different stressors, and if there are conditions under which we may or may not expect them to correlate. In this study, we calculated individual GC/IgA ratios with the assumption that lower GC and higher IgA would be related to better welfare. However, again, there were different responses among camps involved in different activities. For example, elephants that participated in bareback-riding had the highest (Camp D) and lowest (Camp C) overall ratios. This again implies that GC and IgA are affected by more than riding activities, as suggested in other studies [2,10].

There was a seasonal pattern of FGM similar to our previous findings [4,7,74], which was mirrored by changes in salivary cortisol. Reports of seasonal GC production in other Asian elephant populations are mixed; however. In zoo Asian elephants, FGM concentrations did not differ seasonally in the U.S. [75], whereas salivary cortisol was higher between May and October in Spain, although those patterns were highly variable [18]. In semi-captive elephants in Myanmar, elevated FGM concentrations were observed during the logging season (June–August) [76], whereas in Thailand, higher concentrations coincided with the winter high tourist season [4]. Thus, yearly patterns of GCs may be related more to an elephants’ work/activity rather than environmental influences. Moreover, during the high tourist season in Thailand, elephants are fed more high calorie supplements by tourists, which again could lead to increases in GCs [4,7]. That said, cooler temperatures could also create a higher demand for increased metabolism [7,77,78], necessitating greater GC production as reported in other ungulates [79,80].

Annual IgA patterns were not consistent between feces and saliva. Fecal IgA excretion was fairly consistent throughout the year, whereas for saliva, concentrations were clearly lower for the first 5 months of the study, corresponding to the mid- to late-summer season, before increasing in the months leading up to the high tourist season. In humans, Garde et al. [81] reported no annual changes in serum IgA, whereas Weber-Mzel et al. [82] found peak concentrations in winter for salivary IgA, perhaps related to the flu and cold season. Winter is when tourist activity is high in Thai elephant camps, which could be a factor affecting IgA concentrations. However, both sample types showed a high tourist-to-elephant ratio was associated with low, not high, IgA production, so effects on the immune system may not be directly related to numbers of tourists per se. The finding of higher FGM and lower IgA at camps with a higher tourist-to-elephant ratio does warrant further study as it might point to compromised welfare in elephants exposed to too many tourists.

IgA is the first line of defense against viral and bacterial infections [25,27,28], and studies like those of Decaro et al. [26] have suggested a down-regulation of IgA can increase animal susceptibility to disease. Thus, fluctuations in salivary IgA may reveal time windows when elephants are more or less susceptible to problems. Digestive disorders associated with immunodeficiency can be associated with lower IgA levels [83]. According to retrospective records by the National Elephant Institute of Thailand from 1999–2008 [84], the highest prevalence of gastrointestinal tract illnesses in elephants occur during the summer (March–June), which also corresponded with lower salivary IgA concentrations. Finally, in this study, no significant correlations were found between GCs and IgA which was comparable to previous studies in Asian elephants [16,24].

## 5. Conclusions

This was the first study to use IgA in a welfare assessment of elephants participating in different tourist activities. Assuming low GC and high IgA are indicators of better welfare, our overall results showed that this was consistent for only one bareback-riding camp. This may suggest that other factors besides riding affect camp elephant welfare, such as those related to social management, ankus use, chaining, after-hours management, health care, and quality of the mahout–elephant relationship. For example, higher GC/IgA at camps with more tourists per elephant could indicate compromised welfare. The variability in results between saliva and feces for both biomarkers highlights potential differences between acute versus chronic stress responses, perhaps in relation to excretion lag times. It also is important to distinguish between physiological and pathological responses. Therefore, while low GCs often equate with better welfare, it also could be a sign of adrenal exhaustion resulting from chronic stress [85]. Similarly, high IgA may relate to positive welfare states, but it can also be indicative of disease and inflammation [86]. Thus, while IgA may be a useful welfare biomarker in Asian elephants, and the ratio between GC and IgA an informative and novel method for welfare assessments, cautious interpretation is essential. More research is needed to assess individual responses to potential stressors in the tourist environment, the difference between acute and chronic stress responses, and how temperament and coping styles ultimately determine if reactions are adaptive or maladaptive. It is important to note that elephants in this study were selected by camp owners, who perhaps chose animals with a perceived tame disposition because saliva collection is semi-invasive technique. Conversely, selection could also have been guided in part by elephants having a skilled mahout that can get them to participate in untrained behaviors. So while there was no attempt to select lower stress individuals, we cannot rule out that it might have been one outcome of the selection procedure. Regardless, this study provides useful information on additional tools including IgA measures that could be used to assess how management factors affect the welfare of working elephants.

## Figures and Tables

**Table 1:** General information about the six tourist camp facilities in the study, types of activities and husbandry factors, concentrations of glucocorticoids and IgA in feces and saliva, and scores for elephant body condition (1–5; 5 = fattest, 1 = thinnest), foot health (0–3; 0 = no foot and nail problems, 3 = severe problems), and skin wounds (0–2; 0 = no wounds, 2 = major wounds). Means are presented ± SEM.

Variables	Camp ID					
	A	B	C	D	E	F
Activity types	Saddle	Saddle	Bareback	Bareback	No-ride	No-ride
Total number of elephants (M/F)	79 (34/45)	52 (16/36)	19 (1/18)	5 (1/4)	17 (4/13)	47 (13/34)
Number of participating elephants	9	5	9	2	6	13
Numbers of tourists/d (range)	200–300	100–200	20–40	7–15	30–50	50–80
Tourist-to-elephant ratio (range)	2.5–3.8	1.9–3.9	1.1–2.1	1.4–3	1.8–2.9	1.4–2.2
Daily work hours	5–6	5–6	4.5–6	4.5–5	4–6	4–6
Chain hours total (day and night)	17	17	16–18	17–18	17–18	13–18
Chain hours (day only)	3	3	3	3–4	3–4	0–2
Daytime chain length (m)	1.5–2	1.5	1.5	1.5–2	1.5	1.5
Nighttime chain length (m)	2–3	1.5	1.5	3–4	1.5	1.5
Socialization time (h) ^1^	0	0	1	1	1	2–3
Supplemental feeding by tourists	No	No	Yes	Yes	Yes	Yes
FGM (ng/g)	25.8 ± 1.27 ^c^	33.0 ± 1.78 ^a,b,c^	36.30 ± 2.10 ^a,b^	35.65 ± 2.68 ^a,b,c^	31.11 ± 1.72 ^b,c^	39.25 ± 1.72 ^a^
Salivary cortisol (ng/mL)	0.89 ± 0.07 ^a^	0.82 ± 0.06 ^a,b^	0.50 ± 0.03 ^b,c^	0.44 ± 0.07 ^c^	0.71 ± 0.05 ^a,b,c^	0.83 ± 0.05 ^a^
Fecal IgA (µg/g)	0.68 ± 0.09 ^b^	1.26 ± 0.14 ^b^	2.30 ± 0.13 ^a^	0.69 ± 0.14 ^b^	0.67 ± 0.09 ^b^	0.82 ± 0.08 ^b^
Salivary IgA (µg/mL)	5.65 ± 0.52 ^b^	13.49 ± 1.52 ^a^	12.20 ± 1.19 ^a^	3.36 ± 0.41 ^b^	7.57 ± 0.73 ^a,b^	8.00 ± 0.51 ^a,b^
Fecal GC/IgA ^2^	52.34 ± 12.45 ^a,b^	33.69 ± 4.05 ^b^	17.09 ± 2.67 ^b^	101.26 ± 32.06 ^a^	61.94 ± 10.65 ^a,b^	55.01 ± 7.99 ^a,b^
Salivary GC/IgA ^2^	0.26 ± 0.07 ^a^	0.10 ± 0.03 ^a,b^	0.06 ± 0.01 ^b^	0.17 ± 0.05 ^a,b^	0.11 ± 0.02 ^a,b^	0.12 ± 0.03 ^a,b^
Body condition score (1–5)	3.84 ± 0.04 ^a,b^	3.73 ± 0.04 ^b,c^	4.01 ± 0.04 ^a^	3.31 ± 0.08 ^c^	4.09 ± 0.05 ^a^	4.10 ± 0.04 ^a^
Foot score (0–3)	0.89 ± 0.05 ^a,b^	0.98 ± 0.04 ^a^	0.53 ± 0.05 ^b^	0.60 ± 0.10 ^b^	0.85 ± 0.07 ^a,b^	0.90 ± 0.03 ^a^
Wound score (0–2)	0.25 ± 0.04 ^b,c^	0.52 ± 0.07 ^a^	0.07 ± 0.03 ^b,c^	0.31 ± 0.10 ^a,b^	0.06 ± 0.03 ^b,c^	0.03 ± 0.01 ^c^

^a,b,c^ Different superscripts denote statistical differences (*p* < 0.05) across rows for each variable. ^1^ Amount of time elephants were free during the day to socialize with conspecifics. ^2^ Ratios were calculated within the same biological sample and averaged across all elephants at a camp.

**Table 2:** Generalized least square analyses of work activities, season, and camp variables associated with GC and IgA concentrations.

Variables		FGM			Salivary Cortisol			Fecal IgA			Salivary IgA		
		Estimate	SEM	*p*-Value	Estimate	SEM	*p*-Value	Estimate	SEM	*p*-Value	Estimate	SEM	*p*-Value
Age		(DF = 1523; F = 0.15; AIC = 4570.72)			(DF = 1519, F = 0.90; AIC = 957.51)			(DF = 1523; F = 0.24; AIC = 1577.13)			(DF = 1515; F = 4.11; AIC = 3693.48)		
		−0.029	0.07	0.692	0.002	0.002	0.342	0.004	0.009	0.623	0.093	0.046	0.043
Work types		(DF = 2522; F = 9.72; AIC = 4546.44)			(DF = 2518; F = 19.60; AIC = 950.51)			(DF = 2522; F = 15.84; AIC = 1549.68)			(DF = 2514; F = 1.59; AIC = 3686.76)		
	None	Reference											
	Bareback	−0.498	2.144	0.816	−0.299	0.059	<0.01	1.243	0.232	<0.01	2.792	1.597	0.081
	Saddle	−8.267	1.995	<0.01	0.073	0.054	0.177	0.111	0.215	0.604	0.521	1.485	0.725
Camp size		(DF = 2522; F = 0.09; AIC = 4560.92)			(DF = 2518; F = 15.93; AIC = 932.01)			(DF = 2522; F = 6.53; AIC = 1560.53)			(DF = 2514; F = 3.21; AIC = 3682.57)		
	Large	Reference											
	Medium	0.560	2.207	0.799	−0.260	0.052	<0.01	0.803	0.228	<0.01	2.203	1.312	0.093
	Small	2.008	5.014	0.689	−0.409	0.118	<0.01	−0.158	0.522	0.761	−4.831	2.992	0.107
Tourist-to-elephant ratio		(DF = 2522; F = 21.28; AIC = 4533.05)			(DF = 2518; F = 3.13; AIC = 950.88)			(DF = 2522; F = 4.01; AIC = 1565.70)			(DF = 2514; F = 3.36; AIC = 3683.47)		
	High	Reference											
	Moderate	6.745	2.093	<0.01	−0.176	0.087	0.043	0.228	0.323	0.481	3.506	1.761	0.047
	Low	12.254	1.914	<0.01	−0.194	0.079	0.015	0.752	0.295	0.011	4.093	1.601	0.011
Daily work hours		(DF = 1523; F = 13.36; AIC = 4551.26)			(DF = 1519; F = 2.36; AIC = 948.01)			(DF = 1523; F = 0.69; AIC = 1568.63)			(DF = 1515; F = 1.51; AIC = 3687.83)		
		−13.804	3.775	<0.01	0.205	0.133	0.124	0.416	0.498	0.403	3.331	2.706	0.218
Total chain hours		(DF = 1523; F = 15.59; AIC = 4550.82)			(DF = 1519; F = 3.26; AIC = 948.48)			(DF = 1523; F = 2.74; AIC = 1568.75)			(DF = 1515; F = 0.52; AIC = 3690.92)		
	<17 h	Reference											
	≥17 h	−7.556	1.913	<0.01	−0.123	0.07	0.071	0.427	0.258	0.098	1.031	1.418	0.467
Socialization time		(DF = 2522; F = 14.30; AIC = 4540.70)			(DF = 2518; F = 14.97; AIC = 934.44)			(DF = 2522; F = 4.59; AIC = 1564.92)			(DF = 2514; F = 0.55; AIC = 3688.80)		
	0-h	Reference											
	1-h	5.967	1.907	<0.01	−0.296	0.059	<0.01	0.658	0.266	0.013	1.190	1.559	0.445
	2-h	10.834	2.032	<0.01	−0.037	0.063	0.553	−0.066	0.283	0.814	−0.381	−0.229	0.818
Tourist feeding		(DF = 1523; F = 19.84; AIC = 4547.85)			(DF = 1519; F = 8.51; AIC = 943.93)			(DF = 1523; F = 1.81; AIC = 1568.86)			(DF = 1515; F = 0.13; AIC = 3690.54)		
	No	Reference											
	Yes	8.083	1.814	<0.01	−0.184	0.063	<0.01	0.343	0.255	0.178	0.505	1.397	0.717
Months		(DF = 11,513; F = 19.31; AIC = 4367.19)			(DF = 11,509; F = 21.56; AIC = 807.05)			(DF = 11,513; F = 3.82; AIC = 1568.63)			(DF = 11,505; F = 13.41; AIC = 3557.42)		
	January	Reference											
	February	−26.844	3.273	<0.01	0.202	0.102	0.047	0.429	0.202	0.034	0.076	1.530	0.960
	March	−35.979	3.273	<0.01	−0.473	0.102	<0.01	0.348	0.202	0.086	−1.149	1.530	0.453
	April	−30.292	3.273	<0.01	−0.589	0.102	<0.01	0.241	0.202	0.233	−0.419	1.530	0.783
	May	−30.608	3.273	<0.01	−0.406	0.102	<0.01	0.080	0.203	0.693	0.713	1.539	0.643
	June	−37.322	3.273	<0.01	−0.555	0.102	<0.01	0.347	0.202	0.086	2.672	1.539	0.083
	July	−35.958	3.293	<0.01	−0.547	0.103	<0.01	−0.431	0.202	0.033	3.796	1.549	0.014
	August	−32.672	3.293	<0.01	−0.904	0.105	<0.01	−0.262	0.202	0.195	4.999	1.549	<0.01
	September	−32.813	3.273	<0.01	−0.818	0.102	<0.01	−0.157	0.202	0.438	3.980	1.539	0.010
	October	−32.484	3.293	<0.01	−0.810	0.102	<0.01	−0.250	0.202	0.216	1.201	1.549	0.438
	November	−19.914	3.273	<0.01	−0.868	0.102	<0.01	−0.001	0.202	0.995	8.131	1.530	<0.01
	December	−27.003	3.273	<0.01	−0.523	0.102	<0.01	0.246	0.205	0.229	12.519	1.549	<0.01
Environmental season		(DF = 2522; F = 47.39; AIC = 4477.03)			(DF = 2518; F = 32.12; AIC = 897.62)			(DF = 2522; F = 15.69; AIC = 1547.01)			(DF = 2514; F = 15.58; AIC = 3662.10)		
	Rainy	Reference											
	Summer	−0.081	1.792	0.963	0.262	0.059	<0.01	0.531	0.101	<0.01	−3.060	0.854	<0.01
	Winter	15.028	1.792	<0.01	0.472	0.058	<0.01	0.443	0.101	<0.01	1.597	0.854	0.062
Tourist season		(DF = 1523; F = 94.97; AIC = 4478.04)			(DF = 1519; F = 42.73; AIC = 911.24)			(DF = 1523; F = 3.87; AIC = 1568.87)			(DF = 1515; F = 17.89; AIC = 3674.32)		
	High	Reference											
	Low	−15.069	1.546	<0.01	−0.338	0.051	<0.01	−0.178	0.091	0.049	−3.151	0.744	<0.01

**Table 3:** Overall mean fecal and salivary GC to IgA ratios calculated from the same biological sample compared between camp and physical variables.

Variables	Sample	Type	GC/IgA Ratio	*p*-Value
Work activity	Feces	Saddle	37.0 ± 6.0 ^a,b^	<0.01
		Bareback	19.36 ± 2.79 ^b^	
		No-ride	52.68 ± 5.95 ^a^	
	Saliva	Saddle	0.14 ± 0.09	0.066
		Bareback	0.06 ± 0.05	
		No-ride	0.11 ± 0.08	
Camp size	Feces	Small	101.23 ± 32.06 ^a^	0.014
		Medium	21.95 ± 2.37 ^b^	
		Large	42.14 ± 4.64 ^a,b^	
	Saliva	Small	0.17 ± 0.05	0.136
		Medium	0.07 ± 0.01	
		Large	0.13 ± 0.03	
Tourist-to-elephant ratio	Feces	High	52.15 ± 12.45	0.147
		Moderate	39.86 ± 4.99	
		Low	29.13 ± 4.0	
	Saliva	High	0.26 ± 0.07 ^a^	0.015
		Moderate	0.10 ± 0.02 ^b^	
		Low	0.09 ± 0.02 ^b^	
Environmental season	Feces	Summer	22.01 ± 2.34	0.086
		Rainy	36.59 ± 1.84	
		Winter	37.29 ± 7.73	
	Saliva	Summer	0.13 ± 0.02	0.259
		Rainy	0.05 ± 0.01	
		Winter	0.13 ± 0.06	
Tourist season	Feces	High	37.29 ± 7.73	0.269
		Low	29.3 ± 3.08	
	Saliva	High	0.13 ± 0.06	0.370
		Low	0.09 ± 0.02	
Body condition score	Feces	3	53.5 ± 12.66 ^a^	0.041
		4	25.79 ± 3.19 ^b^	
		5	36.85 ± 11.51 ^a,b^	
	Saliva	3	0.27 ± 0.05 ^a^	<0.01
		4	0.11 ± 0.03 ^b^	
		5	0.03 ± 0.01 ^b^	
Foot score	Feces	0	21.92 ± 4.37 ^b^	<0.01
		1	28.25 ± 3.83 ^b^	
		2	66.08 ± 13.74 ^a^	
	Saliva	0	0.09 ± 0.02	0.931
		1	0.10 ± 0.02	
		2	0.1 ± 0.01	
Wound score	Feces	0	23.45 ± 2.78	0.638
		1	26.34 ± 5.57	
	Saliva	0	0.08 ± 0.02	0.838
		1	0.09 ± 0.03	

^a,b^ Values are significantly different within fecal or saliva samples across each variable.

**Table 4:** Association matrix with GC and IgA in saliva and fecal samples.

Parameters	FGM *n* = 525	Salivary Cortisol *n* = 521	Fecal IgA *n* = 525	Salivary IgA *n* = 521
FGM	1.00			
Salivary cortisol	0.14 *	1.00		
Fecal IgA	0.005	0.05	1.00	
Salivary IgA	−0.03	−0.08	−0.04	1.00

* *p* < 0.01.

## Supplementary Materials

The following are available online at https://www.mdpi.com/2076-2615/10/10/1928/s1, Table S1: monthly mean (± SEM) GC and IgA concentrations, Table S2: Age of participating elephants, Figure S1: Monthly fecal glucocorticoid metabolite (FGM; ng/g) and salivary cortisol (ng/ml) concentrations in each individual participating elephant, Figure S2: Monthly fecal (µg/g) and salivary (µg/ml) IgA concentrations in each individual participating elephant.
