# Supplementary material for: Effect of Tourist Activities on Fecal and Salivary Glucocorticoids and Immunoglobulin A in Female Captive Asian Elephants in Thailand
Source: Animals (Basel). 2020 Oct 21;10(10):1928. doi: 10.3390/ani10101928 (PMC7589861; doi:10.3390/ani10101928)
Supplement: Supplementary file 1 [file animals-10-01928-s001.pdf]

Table S1. Summary of monthly mean (± SEM) salivary and fecal glucocorticoid and salivary and fecal immunoglobulin A concentrations throughout the 1-year study in elephant camps offering saddle riding, bareback riding, or no riding.

Month	Saddle				Bareback				Non-ride			
	FGM (ng/g)	Salivary cortisol (ng/ml)	Fecal IgA (μg/g)	Salivary IgA (μg/ml)	FGM (ng/g)	Salivary cortisol (ng/ml)	Fecal IgA (μg/g)	Salivary IgA (μg/ml)	FGM (ng/g)	Salivary cortisol (ng/ml)	Fecal IgA (μg/g)	Salivary IgA (μg/ml)
January	53.10±4.82 (n=14)	1.26±0.15 (n=14)	0.82±0.27 (n=14)	4.62±0.52 (n=14)	75.48±9.64 (n=11)	0.56±0.06 (n=11)	1.65±0.26 (n=11)	6.05±0.42 (n=11)	61.64±4.10 (n=19)	1.67±0.17 (n=19)	0.93±0.23 (n=19)	6.33±0.61 (n=19)
February	25.75±5.25 (n=14)	1.36±0.17 (n=14)	1.47±0.37 (n=14)	5.38±0.57 (n=14)	48.48±3.38 (n=11)	1.19±0.18 (n=11)	2.46±0.39 (n=11)	5.97±0.75 (n=11)	35.26±2.89 (n=19)	1.70±0.13 (n=19)	0.97±0.22 (n=19)	5.99±0.58 (n=19)
March	22.37±2.51 (n=14)	1.15±0.40 (n=14)	0.83±0.24 (n=14)	4.10±0.64 (n=14)	33.08±5.51 (n=11)	0.29±0.09 (n=11)	2.59±0.33 (n=11)	4.46±0.58 (n=11)	25.51±2.92 (n=19)	0.81±0.14 (n=19)	1.18±0.24 (n=19)	4.97±0.52 (n=19)
April	24.47±2.00 (n=14)	0.69±0.08 (n=14)	1.66±0.48 (n=14)	3.81±0.77 (n=14)	38.14±3.47 (n=11)	0.36±0.09 (n=11)	5.22±0.57 (n=11)	4.63±0.56 (n=11)	34.20±4.16 (n=19)	0.85±0.09 (n=19)	0.38±0.13 (n=19)	6.78±0.86 (n=19)
May	25.89±2.17 (n=14)	1.30±0.16 (n=14)	0.97±0.24 (n=14)	5.18±0.96 (n=14)	27.16±2.71 (n=11)	0.53±0.07 (n=11)	1.77±0.25 (n=11)	6.74±0.85 (n=11)	38.79±4.01 (n=19)	0.71±0.07 (n=18)	0.94±0.28 (n=18)	7.21±0.71 (n=18)
June	21.72±1.81 (n=14)	0.76±0.05 (n=14)	0.59±0.20 (n=14)	9.33±1.47 (n=14)	24.07±1.98 (n=11)	0.58±0.07 (n=11)	3.45±0.64 (n=11)	8.82±1.70 (n=10)	28.09±1.66 (n=19)	0.74±0.04 (n=19)	1.48±0.36 (n=19)	7.33±1.09 (n=19)
July	22.53±2.30 (n=13)	0.99±0.18 (n=14)	0.58±0.14 (n=14)	8.96±3.56 (n=14)	21.13±1.46 (n=11)	0.47±0.05 (n=11)	0.88±0.30 (n=11)	13.47±3.18 (n=11)	32.31±1.87 (n=19)	0.64±0.05 (n=17)	0.55±0.15 (n=19)	7.71±1.63 (n=18)
August	24.94±2.43 (n=13)	0.47±0.09 (n=14)	0.71±0.14 (n=14)	9.83±2.64 (n=14)	28.84±3.72 (n=11)	0.31±0.05 (n=9)	1.50±0.23 (n=11)	17.43±3.87 (n=9)	33.79±3.69 (n=19)	0.30±0.04 (n=17)	0.49±0.16 (n=19)	7.40±1.16 (n=17)
September	41.83±3.08 (n=14)	0.51±0.04 (n=14)	0.48±0.14 (n=14)	14.60±2.57 (n=14)	26.93±2.99 (n=11)	0.45±0.06 (n=11)	2.08±0.44 (n=11)	10.85±1.72 (n=11)	22.06±2.07 (n=19)	0.40±0.04 (n=19)	0.57±0.19 (n=19)	5.17±0.78 (n=18)
October	24.21±2.13 (n=14)	0.56±0.14 (n=14)	0.55±0.14 (n=14)	5.95±1.41 (n=14)	36.15±4.13 (n=10)	0.34±0.06 (n=11)	2.00±0.30 (n=11)	6.10±1.48 (n=11)	30.87±3.20 (n=19)	0.44±0.05 (n=19)	0.50±0.13 (n=19)	8.10±1.49 (n=19)
November	26.35±1.91 (n=14)	0.53±0.16 (n=14)	1.04±0.33 (n=14)	14.55±4.21 (n=14)	39.05±5.97 (n=11)	0.24±0.04 (n=11)	1.54±0.34 (n=11)	19.50±6.57 (n=11)	56.33±7.76 (n=19)	0.39±0.06 (n=19)	0.83±0.30 (n=19)	10.05±2.26 (n=19)
December	27.15±1.67 (n=14)	0.81±0.08 (n=14)	1.23±0.27 (n=14)	14.17±3.18 (n=14)	35.68±5.75 (n=11)	0.56±0.09 (n=11)	2.53±0.50 (n=9)	25.25±6.57 (n=10)	41.27±4.24 (n=19)	0.79±0.06 (n=19)	0.71±0.16 (n=19)	17.70±2.26 (n=18)

Table S2 Age of participating elephants

Age	Number of participating elephants		
	**Saddle**	**Bareback**	**No-riding**
1-10		1	2
11-20		2	5
21-30	5	3	2
31-40	3	1	6
41-50	6	3	3
51-60		1	1

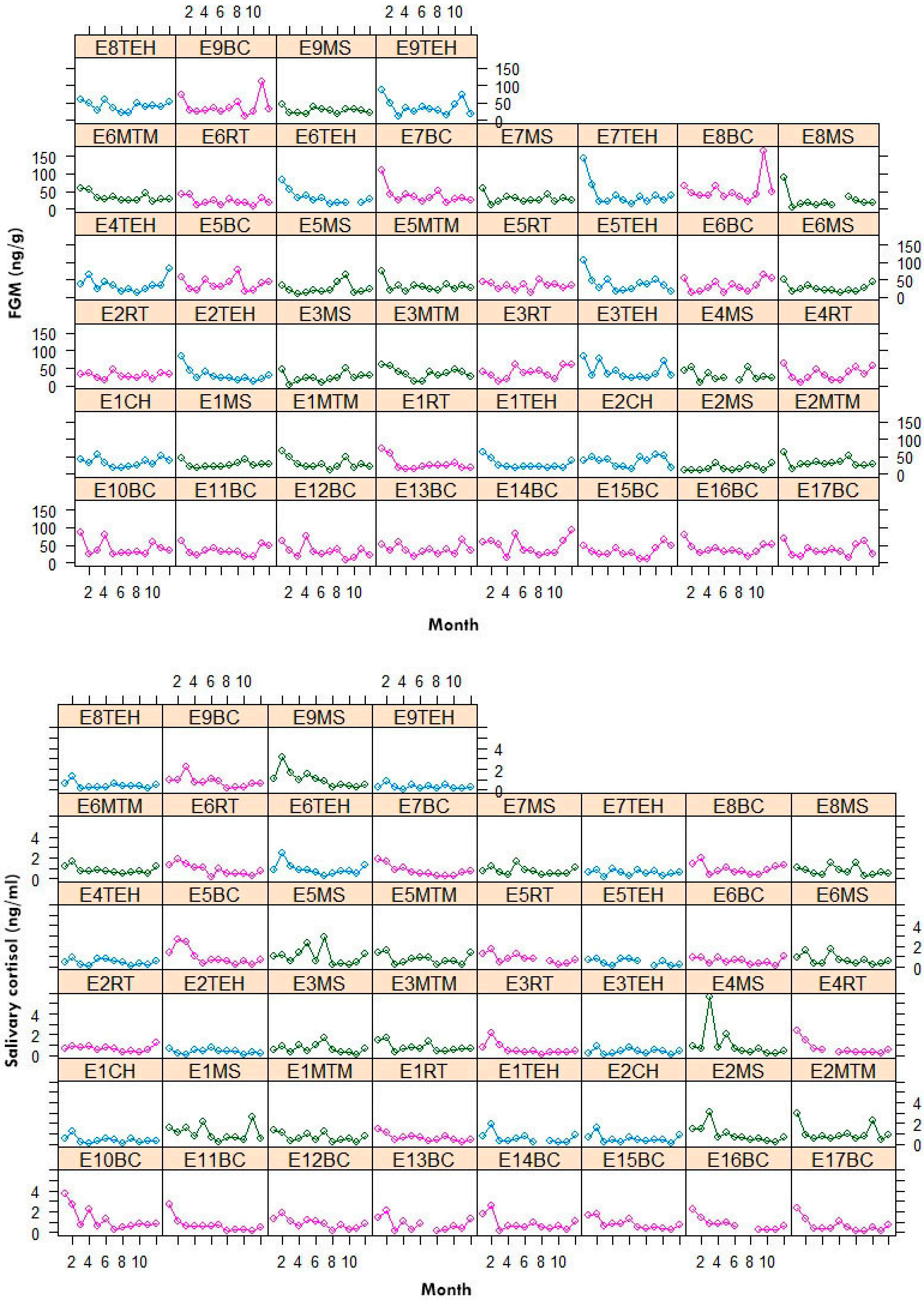

Figure S1. Monthly fecal glucocorticoid metabolite (FGM; ng/g) and salivary cortisol (ng/ml) concentrations in each individual participating elephant.

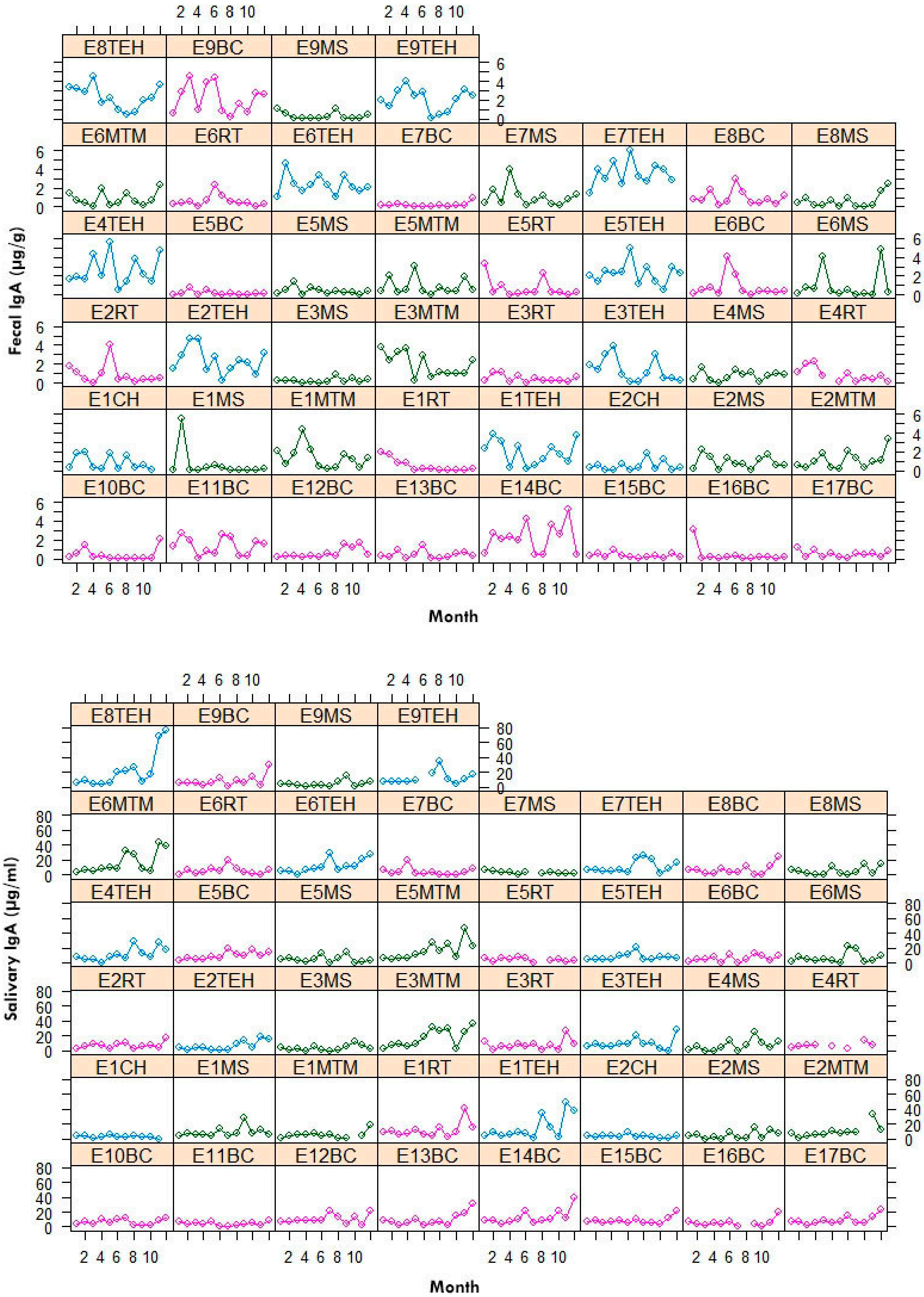

Figure S2 Monthly fecal (µg/g) and salivary (µg/ml) IgA concentrations in each individual participating elephant.
